# Cerebral Palsy, COVID-19, and Neurolipidosis in an 18-Year-Old Female

**DOI:** 10.7759/cureus.18294

**Published:** 2021-09-26

**Authors:** George S Stoyanov, Deyan L Dzhenkov, Hristo Popov, Emran Lyutfi, Lilyana Petkova

**Affiliations:** 1 General and Clinical Pathology/Forensic Medicine and Deontology, Medical University of Varna, Varna, BGR; 2 Neurological Diseases and Neuroscience, Medical University of Varna, Varna, BGR

**Keywords:** neimann-pick disease, autopsy, histopathology, neurolipidosis, cerebral palsy, covid-19

## Abstract

Since the novel coronavirus (COVID-19) pandemic started, children and young adults have seldom been placed in high-risk groups, despite reports that they are at increased risk of severe forms of the disease and death in the presence of comorbidities. Herein we report an autopsy case of an 18-year-old female with a history of cerebral palsy (CP), recurrent respiratory infections, and newly diagnosed COVID-19, and who expired 22 days after presenting with symptoms of the disease. Gross findings were concurrent with CP-significant hypotrophy, with deep and wide brain sulci. The lungs grossly were with increased weight and blood-filled. Histopathology of the respiratory system showed the well-established COVID-19-associated alveolar multinucleated cells, type two pneumocyte hyperplasia, and vascular changes. Furthermore, foci of groups of enlarged cells with foamy cytoplasm were identified in the pulmonary interstitium. Similar changes were also seen in the spleen, liver, and central nervous system, concurrent with an unrecognized lipid storage disease. The clinically unrecognized neurolipidosis, corresponding morphologically and clinically to Niemann-Pick disease type B, leading to interstitial lung disease and recurrent respiratory infections, inevitably played a role in the severity and progression of COVID-19 in our case, despite the age.

## Introduction

Ever since the emergence of the severe acute respiratory distress syndrome coronavirus 2 (SARS-CoV-2) causing the novel coronavirus disease (COVID-19), alarms have been raised on the presence of severe clinical complications in patients with multiple morbidities and malnutrition [1-3]. Despite initial reports that clinical symptoms and disease progression in children and young adults are less severe, research has highlighted the potential severity of the infection in these populations [4-7]. Alarms have also been raised for the possible severe clinical course in young patients with chronic conditions, despite the minimal mortality reported in this group [7,8].

Herein we report a case of an 18-year-old caucasian female with cerebral palsy (CP), a history of recurrent respiratory infections, and a newly diagnosed SARS-CoV-2 infection.

## Case presentation

Clinical history

The patient was born from a first, unfollowed, high-risk pregnancy at home. Birth weight was 2400 grams. Due to a severely depressed state, medical attention was sought with the establishment of severe perinatal hypoxia. Oxygen treatment was started, but there was an onset of neurological symptoms with limb hypotonia and hyporeflexia. A pediatric neurologist was consulted, and the diagnosis of cerebral palsy (CP) was established. Treatment with physiotherapy was started.

The patient's condition did not show improvement and developed severe peripheral atrophy and large joint contractures, severe malnutrition, cognitive and developmental delay. Due to the malnutrition, a gastrostomy was placed, but weight gain did not improve.

The child had multiple hospitalizations per year, predominantly with recurrent respiratory infections. The last hospitalization before the current one, establishing a pulmonary abscess with Pseudomonas aeruginosa as the culprit. The patient responded well to antibiotic treatment and was discharged on day 12 without any pulmonary symptoms.

Current hospitalization

Several days after the last discharge, the patient had an elevated body temperature of 39 degrees celsius and developed a dry cough. An outpatient COVID-19 rapid antigen test gave a positive result and the patient was transferred for hospital treatment due to her severe concomitant conditions.

An in-hospital real-time polymerase chain reaction (RT-PCR) test for COVID-19 also came back positive and a thoracic x-ray revealed bilateral changes in the lungs. Despite complete COVID-19 protocols being initiated, the patient's condition steadily deteriorated, with the development of hypoxia (oxygen saturation 80%). Multiple repeat RT-PCRs on days five, ten, and fifteen once again came back positive and the patient expired on day 22 of hospitalization.

Gross findings

The patient was severely hypotrophic, with height measuring at 150cm, weight at 19kg and body mass index (BMI) of 8.4kg/m2 (severely underweight), head circumference at 47cm. Contractions of the large joints of the upper and lower limb were present and a gastrostomy tube in the epigastrium.

The organ section showed obliteration of the upper part of the pleural cavity on the right side, corresponding to the area of pulmonary abscess in the previous hospitalization. The mucosa of the trachea and bronchi was edematous and hyperemic, with areas of purulent deposits in the small bronchi. The lungs were enlarged, deformed, heavy (left lung 400g at 2.1% of body weight, right lung 550g at 2.9% of body weight) and blood-filled, with minimal preserved air-filled areas, from which edematous fluid discharged spontaneously.

The heart was enlarged (120g, 0.63% of body weight) with a left ventricular thickness of 9mm and right ventricular thickness of 3mm. No other significant gross changes were noted throughout the remainder of the cardiovascular system.

The central nervous system (CNS) weighed 870g (4.58% of body weight), with edematous gyri and deep and wide sulci, most pronounced in the cerebellum. Mild hepatosplenomegaly was present, without grossly visible changes on the parenchyma of the liver (900g, 4.74% of body weight) and spleen (200g, 1.05% of body weight). The remaining systems did not show any gross changes, apart from the skeletal muscles, which were severely atrophic (Table 1).

**Table 1:** organ weight and correlation to total body mass Note: Despite the low weight of the liver and central nervous system, when compared to the rest of the organs and represented as a percentage of total body weight, they are significantly increased

Organ	Weight (g)	% of total body weight
Left lung	400	2.1
Right lung	550	2.9
Heart	120	0.63
Central nervous system	870	4.58
Liver	900	4.74
Spleen	200	1.05
Left kidney	100	0.53
Right kidney	90	0.47

Histopathology

**Figure 1:**
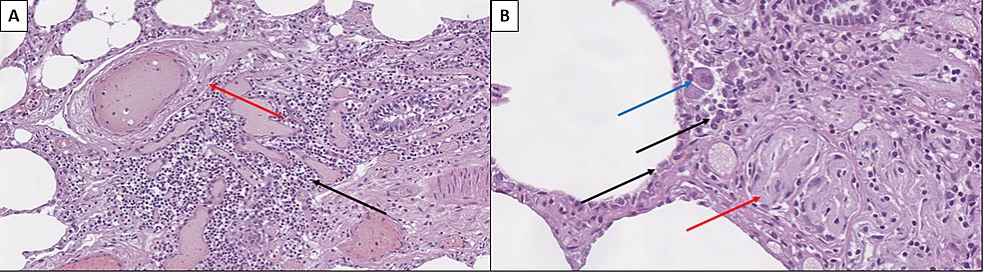
Pulmonary histopathology in COVID-19 A: Interstitial lymphoplasmacytic inflammatory infiltration (black arrow) and severe vascular wall edema of small arteries (red arrow), hematoxylin and eosin stain, original magnification 200x. B: Type two pneumocyte hyperplasia (black arrows), alveolar cell multinucleation (blue arrow) and interstitial fibrosis, hematoxylin and eosin stain, original magnification 400x.

**Figure 2:**
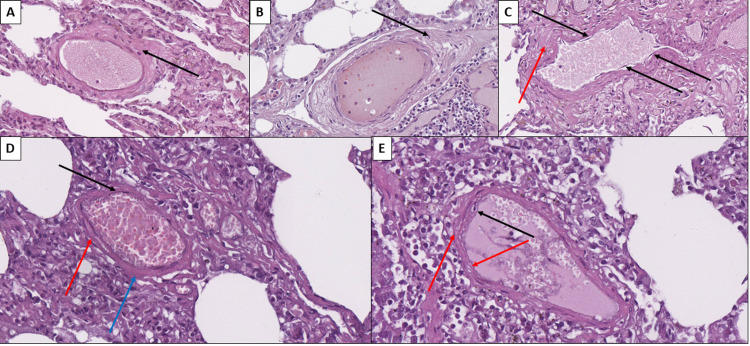
Histopathology of pulmonary blood vessel changes associated with COVID-19 A: Subendothelial vascular wall deposits (arrow) in arteriole, hematoxylin, and eosin stain, original magnification 400x. B: Vascular wall edema (arrow) in a small artery, hematoxylin and eosin stain, original magnification 400x. C: Endotheliitis - endothelial delamination (black arrows) and subendothelial deposits (red arrow), hematoxylin and eosin stain, original magnification 400x. D: Degenerative endothelial changes with metachromasia (black arrow), subendothelial edema (red arrow), and hyaline-like degeneration of part of the vascular wall (blue arrow) in arteriole, hematoxylin and eosin stain, original magnification 400x. E: Endothelial cell edema/ballooning (black arrow) and subendothelial edema (red arrows), hematoxylin and eosin stain, original magnification 400x.

Histopathology of the lungs revealed COVID-19-associated changes (Figures 1 and 2). These included the well-established presence of multinucleated interalveolar cells, type two pneumocyte hyperplasia, as well as endotheliitis, with subendothelial deposits in the vascular walls and vascular wall degeneration as seen in Figures 1 and 2.

**Figure 3:**
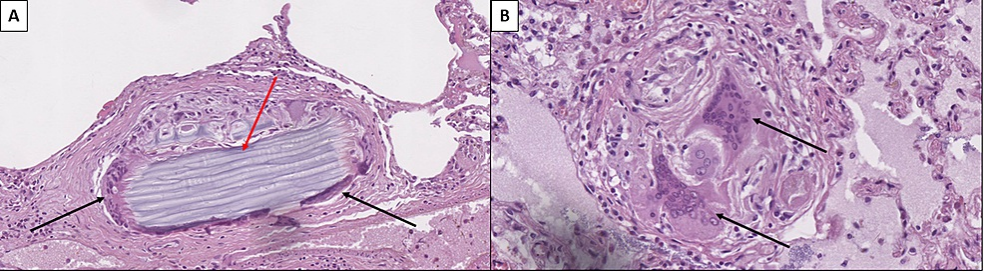
Aspiration of foreign body A: Foreign body granuloma with multinucleated giant cells (black arrows) and foreign body in the center of the granuloma (red arrow), hematoxylin and eosin stain, original magnification 200x. B: Foreign body granuloma with multinucleated giant cells (black arrows) and complete resorption of the foreign body, hematoxylin and eosin stain, original magnification 400x.

Other non-COVID-19-associated inflammatory changes in the pulmonary parenchyma were foci of purulent bronchitis and pulmonary foreign bodies, with granuloma formation (Figure 3). These changes corresponded to a superimposed bacterial infection within the respiratory system, a common phenomenon in the setting of viral infection, as well as multiple severe episodes of aspiration.

**Figure 4:**
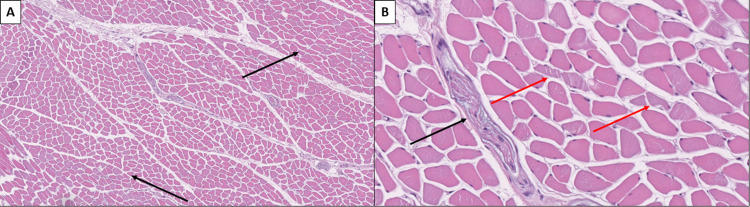
Skeletal muscle and peripheral nerve atrophy A: Groups of degenerative rhabdomyocytes (black arrows), hematoxylin and eosin stain, original magnification 100x. B: Peripheral nerve with Wallerian degeneration (black arrow) and degenerative rhabdomyocytes (red arrows), hematoxylin and eosin stain, original magnification 400x.

Skeletal muscles showed severe muscle fiber atrophy and Wallerian degeneration of peripheral nerves (Figure 4).

**Figure 5:**
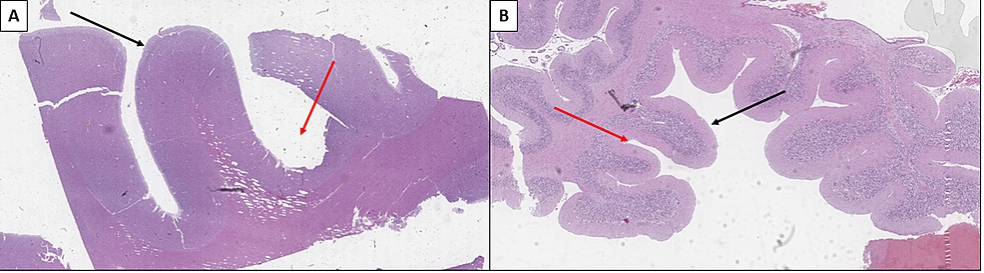
Atrophic changes of the central nervous system A: Atrophic cerebellum with small gyri (black arrow) and wide sulci (red arrow), hematoxylin and eosin stain, original magnification 10x. B: Atrophic cerebellum with small gyri and shallow and wide sulci, hematoxylin and eosin stain, original magnification 20x.

The CNS showed myriad changes with atrophic gyri and shallow and wide sulci - the histological substrate of CP, as well as cerebral edema (Figure 5).

**Figure 6:**
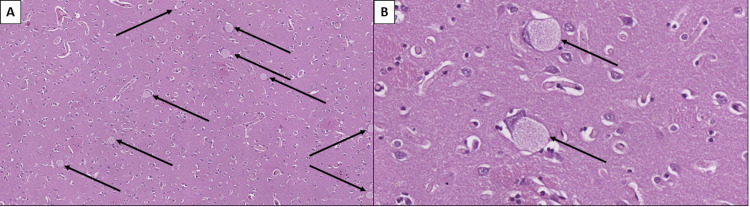
Cerebral neurolipidosis A: Multiple enlarged neurons with foamy cytoplasm (arrows), hematoxylin and eosin stain, original magnification 100x. B: Enlarged neurons with an eccentrically displaced nucleus and foamy cytoplasm (arrows), hematoxylin and eosin stain, original magnification 400x.

Furthermore, multiple cortical areas and the brainstem showed aggregation of enlarged neurons, with an eccentrically located flattened nucleus and foamy cytoplasm (Figure 6).

**Figure 7:**
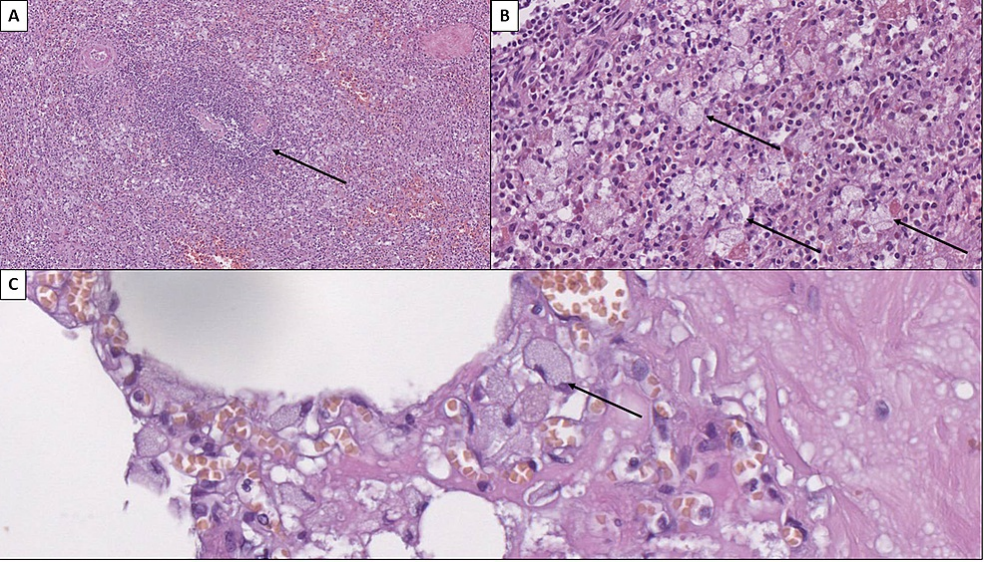
Aggregation of enlarged cells with foamy cytoplasm A: Preserved splenic corpuscle (arrow) with aggregation of foamy cells around it, hematoxylin and eosin stain, original magnification 100x. B: Aggregation of groups of enlarged cells with foamy cytoplasm (arrows) in the red pulp of the spleen, hematoxylin and eosin stain, original magnification 400x. C: Aggregation of groups of enlarged cells with foamy cytoplasm in the pulmonary interstitium (arrow), hematoxylin and eosin stain, original magnification 400x.

Similar findings with large cells with foamy cytoplasm were also identified within the liver, spleen, and lung interstitium, where they were clustered into small groups (Figure 7). These cells, across all organs, did not show a histochemical reaction with the Periodic acid-Schiff (PAS) stain but reacted with lipid-specific histochemical stains such as Oil red and Sudan black stain. These histochemical reactions showed that the aggregation within the cells was lipid in nature. Due to the organ-specific sites of aggregation, these correlated to cells of accumulation in a lipid storage disease.

As no evidence of acute respiratory distress syndrome was detected, the protocol was signed off as CP, neurolipidosis (with undetected enzyme deficient), and COVID-19 infection with severe pulmonary changes, leading to hypoxia, acute respiratory and cardiovascular failure with cerebral edema, and herniation. Despite no enzyme or genetic testing being performed, the condition in question is closest to the morphology and clinical manifestations of Niemann-Pick disease type B.

## Discussion

Concomitant morbidity is an important factor for the course of COVID-19 infection in young patients [8]. In the described patient, both the clinically recognized and unrecognized neurological diseases played an essential role in the progression of the infection as they have both been described to have significant pulmonary complications [9-14].

Lipid storage diseases are a group of genetic conditions, leading to enzymatic defects with the accumulation of intermediate metabolic lipid products in the tissues of the organism [12, 15]. Neurolipidoses are a subset of lipid storage diseases in which the CNS is one of the affected organs [15].

The clinically unrecognized neurolipidosis in our case, despite the lack of enzyme or genetic testing, is closest to Niemann-Pick disease, type B [12,13,15]. Our differential diagnosis on the histological findings in the CNS, spleen, liver, and pulmonary interstitium included Niemann-Pick types A and C as well as Krabbe and Gaucher disease. However, the patient profile, histopathology, and organ involvement did not fit these conditions [16-18].

Type C Niemann-Pick disease is especially unlikely in our case due to aggregated evidence that viral particles cannot properly interact with the intracellular domain due to mutations in the Niemann-Pick type C intracellular cholesterol transporter 1 (NPC1) [19]. The presence of mutant forms of NPC1 inhibits the viral entry and infection by decreasing the quantity and stability of angiotensin-converting enzyme 2 (ACE2) on the cell membrane as well as causing abnormalities in the location and function of cathepsin L, altogether which decrease the chance of viral fusion and replication, basically locking the viral particles in the lysosomes [20]. Furthermore, NPC1 mutant cells have increased levels of oxysterols 25-hydroxycholesterol (25-HC) and 7-ketocholesterol (7-KC), which also have antiviral effects [20]. A similar mechanism of cellular mechanisms has been described in Ebola and several other viral infections [19,20]. As such, several drugs which can induce an NPC1 phenotype in the cell, such as chloroquine, triazole antifungals, and others, have been proposed to have possible anti-COVID-19 effects [20].

Clinically, despite the multiple neuropediatric evaluations, the CNS involvement was interpreted as aggregated changes from the severe form of CP [9,12]. The recurrent and progressive respiratory symptoms were also unrecognized as part of the condition. They were interpreted as a result of the chronic aspirations, as seen in Figure 3, due to the CP alone [9,12].

Despite the CP and neurolipidosis in our patient not being directly connected to the SARS-CoV2 infection, the pulmonary changes associated with these conditions inevitably played a role in COVID-19 progression and severity [3, 9, 10, 13, 14].

## Conclusions

Children and young adults remain at high risk of severe COVID-19 and death, especially in the context of severe comorbidities, as in the presented case. Congenital diseases and malformations, malignancy and obesity remain amidst the most common and severe concomitant conditions and predictive factors for COVID-19 outcome in these populations.

Despite Niemann-Pick type C being proposed as a disease where the intracellular migration of viral particles is impeded, as shown here, other forms of neurolipidoses diseases do not seem to hinder viral intracellular pathways. These are due to the specifics of the intracellular defect and the pathways the virus needs for its penetration, integration and replication in the cells.

Furthermore, severe neurological impediments, hepatic and splenomegaly, should always require the exclusion of lipid storage diseases, even in the presence of a previously determined condition.
